# Radiation reduction for interventional radiology imaging: a video frame interpolation solution

**DOI:** 10.1186/s13244-024-01620-z

**Published:** 2024-02-14

**Authors:** Zhijiang Tang, Qiang Xiong, Xuantai Wu, Tianyi Xu, Yuxuan Shi, Ximing Xu, Jun Xu, Ruijue Wang

**Affiliations:** 1 School of Statistics and Data Science, Nankai University, Tianjin, China; 2 Department of Hepatobiliary Surgery Children’s Hospital of Chongqing Medical University, National Clinical Research Center for Child Health and Disorders, Ministry of Education Key Laboratory of Child Development and Disorders, Chongqing Key Laboratory of Structural Birth Defect and Reconstruction, Children’s Hospital of Chongqing Medical University, Chongqing, China; 3 Big Data Engineering Center, Children’s Hospital of Chongqing Medical University, National Clinical Research Center for Child Health and Disorders, Ministry of Education Key Laboratory of Child Development and Disorders, Chongqing Key Laboratory of Structural Birth Defect and Reconstruction, Children’s Hospital of Chongqing Medical University, Chongqing, China

**Keywords:** Interventional radiology imaging, Radiation exposure reduction, Deep learning, Radiation safety

## Abstract

**Purpose:**

The aim of this study was to diminish radiation exposure in interventional radiology (IR) imaging while maintaining image quality. This was achieved by decreasing the acquisition frame rate and employing a deep neural network to interpolate the reduced frames.

**Methods:**

This retrospective study involved the analysis of 1634 IR sequences from 167 pediatric patients (March 2014 to January 2022). The dataset underwent a random split into training and validation subsets (at a 9:1 ratio) for model training and evaluation. Our approach proficiently synthesized absent frames in simulated low-frame-rate sequences by excluding intermediate frames from the validation subset. Accuracy assessments encompassed both objective experiments and subjective evaluations conducted by nine radiologists.

**Results:**

The deep learning model adeptly interpolated the eliminated frames within IR sequences, demonstrating encouraging peak signal-to-noise ratio (PSNR) and structural similarity index (SSIM) results. The average PSNR values for angiographic, subtraction, and fluoroscopic modes were 44.94 dB, 34.84 dB, and 33.82 dB, respectively, while the corresponding SSIM values were 0.9840, 0.9194, and 0.7752. Subjective experiments conducted with experienced interventional radiologists revealed minimal discernible differences between interpolated and authentic sequences.

**Conclusion:**

Our method, which interpolates low-frame-rate IR sequences, has shown the capability to produce high-quality IR images. Additionally, the model exhibits potential for reducing the frame rate during IR image acquisition, consequently mitigating radiation exposure.

**Critical relevance statement:**

This study presents a critical advancement in clinical radiology by demonstrating the effectiveness of a deep neural network in reducing radiation exposure during pediatric interventional radiology while maintaining image quality, offering a potential solution to enhance patient safety.

**Key points:**

• Reducing radiation: cutting IR image to reduce radiation.

• Accurate frame interpolation: our model effectively interpolates missing frames.

• High visual quality in terms of PSNR and SSIM, making IR procedures safer without sacrificing quality.

**Graphical Abstract:**

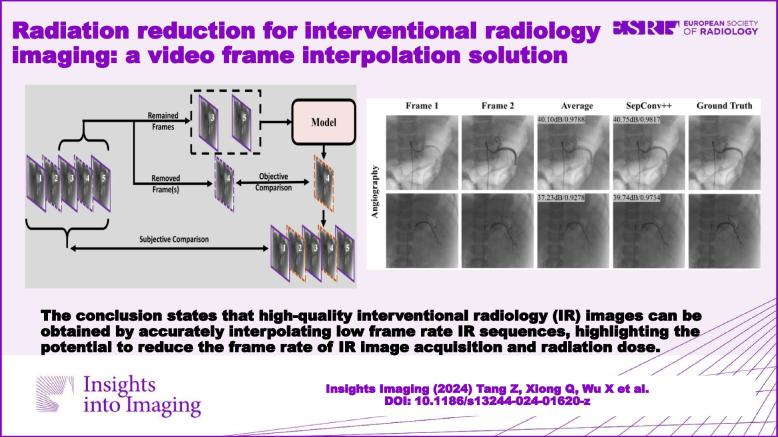

## Introduction

Interventional radiology (IR) imaging stands as the gold standard for diagnosing vascular diseases [1]. The field has undergone substantial development in tandem with advancements in endovascular techniques over the past decades [2]. Nonetheless, the procedure exposes both patients and interventionalists to ionizing radiation, inherently posing health risks. The intricate anatomical structures and procedural intricacies magnify radiation exposure, escalating these risks [3]. Notably, children often display heightened radiosensitivity in comparison to adults, resulting in prolonged survival times post-radiation exposure and an extended duration of radiological cancer risk. Consequently, pediatric patients often encounter more severe radiation-related risks than adults [4]. The aim of our study is to explore a new potential for reducing radiation dosage in interventional radiology.

The IR protection guidance established by the German Association of Physicians [5] outlines various strategies for mitigating radiation exposure in IR imaging. Firstly, reducing the radiation dose is achievable through real-time digital fluoroscopy procedures and the adoption of lower frame rates [6]. Secondly, shortening exposure time can be accomplished by implementing pulsed fluoroscopy rather than continuous fluoroscopy during IR examinations [5], or by increasing the distance between the patient and the radiation source [7]. Additionally, protective shielding, whether the facility level [7] or individual level as exemplified by [5], plays a crucial role in shielding patients and interventional personnel from radiation exposure. However, there is still a lack of research on optimizing critical parameters like voltage, current, exposure time, and frame rate [2], to effectively reduce radiation in IR imaging while maintaining the visual quality of IR sequences necessary for clinical diagnosis.

To minimize radiation exposure for patients and interventionalists, an optimal balance can be achieved between the frame rate of the acquired IR sequence and the radiation dose through the selection of an appropriate acquisition mode. Dose per frame and frame rate (frames per second, or FPS) are crucial parameters in IR imaging systems. Improving IR imaging quality often necessitates an increase in either the dose per frame or frame rate, consequently leading to higher radiation exposure. Lower IR frame rates are commonly favored as the default setting. However, low-frame-rate IR sequences frequently display flicker artifacts, potentially compromising an interventionalist’s assessment of intricate blood flow and conditions [3, 8].

We are encouraged by substantial advancements in artificial intelligence and deep learning techniques in recent years. These advancements have notably contributed to progress in video enhancement tasks and medical image analysis [9–11]. However, research on frame interpolation of IR sequences for radiation dose reduction is still in its early stage, with limited research [12–15] exploring deep learning approaches on DSA sequences without significant motion. There are also studies proposing interleaving high-dosage frames with low-dosage ones to acquire two types of sequences simultaneously, thereby reducing radiation dosage. Other deep learning research in DSA primarily focuses on the generation of subtraction sequences, which only a few addressing the radiation dosage problem. The aim of this study is to develop and validate an effective deep learning model enabling to sample digital subtraction angiography (DSA) images at low frame rates. This allows for reduced radiation dosage while ensuring high-quality IR images meeting the clinical requirements can still be restored. To the best of our knowledge, our study is the first to explore this application on IR images with noticeable motions and be validated by both subjective and objective experiments. This innovation holds the potential in facilitating the implementation of pediatric interventional radiology and mitigating the radiation risks.

## Materials and methods

### Data collection and processing

A retrospective collection involved 1634 IR image sequences obtained from 167 children who underwent examination and treatment at the Children’s Hospital of Chongqing Medical University from March 12, 2014, to January 7, 2022. The study was registered in the Chinese Clinical Trial Registry (ChiCTR2200058971) and conducted in compliance with the Declaration of Helsinki (DoH), with approval from the institutional ethical review board (File No. 2022, 69). Informed consent was waived.

To ensure the model’s accurate learning of blood flow interpolation, we excluded abnormal IR sequences displaying blurry motions caused by camera or human movement, as well as static sequences lacking blood flow [16]. Additionally, IR sequences without breath motion, providing limited information on vascular motion, were also omitted. Consequently, after data preprocessing, a total of 1367 sequences comprising 95,308 images were retained. These sequences encompassed arterial, venous, and portal vein angiography imaging across various anatomical regions.

**Fig. 1:**
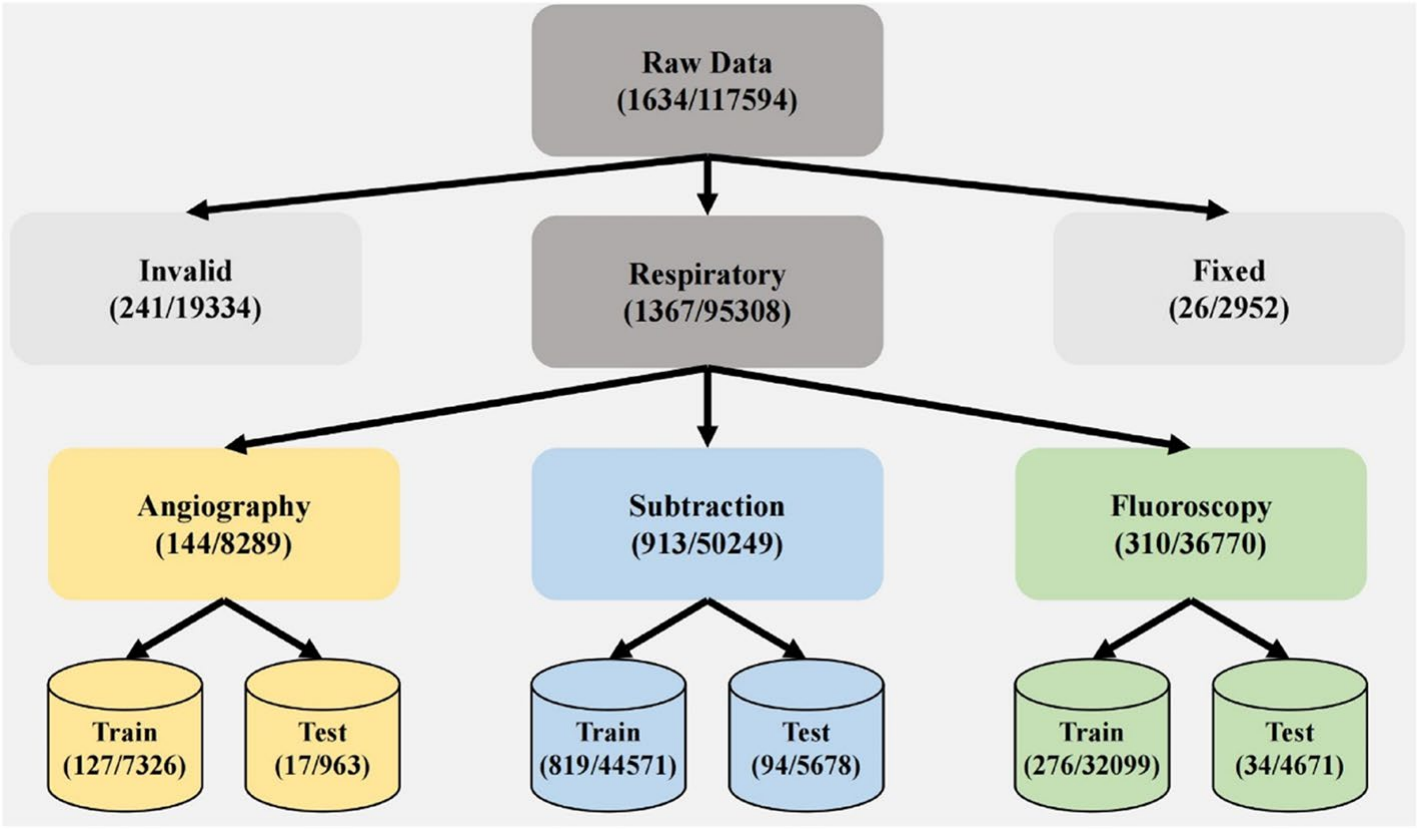
Illustration of our data pre-processing stage. The raw data are firstly classified into invalid data, data with breath motion (respiratory), and data without breath motion (fixed). Then the sequences with breath motion are divided into three modalities, e.g., angiography, subtraction, and fluoroscopy, for model training and testing. The amounts of sequences and images are presented accordingly at each stage

The IR sequences encompass three modalities: angiography, subtraction, and fluoroscopy. Each modality had 90% of its sequences randomly assigned to the training set, while the remaining 10% allocated to the test set. Angiography sequences, directly acquired form IR imaging equipment without processing, typically exhibited frame rates from 4 to 10 FPS and image sizes ranging from 500 × 500 to 1200 × 900. Subtraction sequences, derived from angiography, eliminated bone and soft-tissue information to emphasize blood vessel visualization [16]. Image sizes of subtraction sequences typically ranged from 200 × 200 to 500 × 500. Fluoroscopy sequences, recorded by physicians during DSA surveillance, provided limited vascular information, having frame rates from 7 to 15 FPS and image sizes from 100 × 100 to 900 × 900. Table 1 summarizes detailed information about the data sequences and patient characteristics. The high-frame-rate IR sequences obtained were considered as the original sequences in the training and test sets. To create corresponding low-frame-rate IR sequences, every alternate frame was excluded, while always retaining the first frame, from each high-frame-rate IR sequence. The overall data preprocessing workflow is illustrated in Fig. 1.

**Table 1:** Summary of different group data sequences and patient characteristics. Data were provided by the Children’s Hospital of Chongqing Medical University. Information in each line shows the number (percentage) or the mean (standard deviation) values

	**Angiography train**	**Angiography test**	**Subtraction train**	**Subtraction test**	**Fluoroscopy train**	**Fluoroscopy test**
**Patient #Average age** (in years)	27	14	148	63	58	26
Range	(0.58–15)	(0.58–13)	(0.25–15)	(0.58–14)	(0.25–15)	(0.25–15)
**Sex**						
Female	16 (59.3%)	9 (64.3%)	63 (42.6%)	23 (36.5%)	32 (36.5%)	15 (57.7%)
Male	11 (40.7%)	5 (35.7%)	85 (57.4%)	40 (63.5%)	26 (44.8%)	11 (42.3%)
**Sequence # FPS**	127	17	819	94	276	34
3 FPS	—	1 (59%)	—	—	—	—
4 FPS	92 (72.4%)	11 (64.7%)	731 (89.3%)	82 (86.3%)		
6 FPS	11 (8.7%)	2 (11.8%)	15 (1.8%)	2 (2.1%)	—	—
7 FPS	—	—	6 (0.7%)	1 (1.1%)	52 (18.8%)	6 (17.6%)
10 FPS	24 (18.9%)	3 (25.6%)	46 (5.6%)	6 (6.3%)	170 (61.6%)	20 (58.8%)
15 FPS	—	—	21 (2.6%)	3 (3.2%)	46 (16.7%)	6 (17.6%)
30 FPS	—	—	—	—	8 (2.9%)	2 (5.9%)
**Images**	7326	963	44,571	5678	32,099	4671

### Deep learning model for IR sequence interpolation

We utilized the SepConv +  + deep learning model [8] for IR frame interpolation due to its promising performance in video frame interpolation tasks. SepConv +  + is a lightweight model with less than 20 million parameters and capable of processing 5 to 20 frames per second. It employs a U-Net-style backbone [17] which initially contracts into small-scale feature maps using convolutions and then expands to the original size while preserving detailed information through skip connections. SepConv +  + extracts features to estimate two sets of convolution kernels that specify motion between the frames. These kernels were applied in the convolution process of preceding and succeeding frames, and their output features were fused to generate the interpolated intermediate frame. Training was conducted separately on the training set for each IR modality and on a combined training set comprising sequences from all three IR modalities. Implemented in PyTorch [18], SepConv +  + was executed on an NVIDIA RTX 3060 GPU.

### Model validation and statistical analysis

**Fig. 2:**
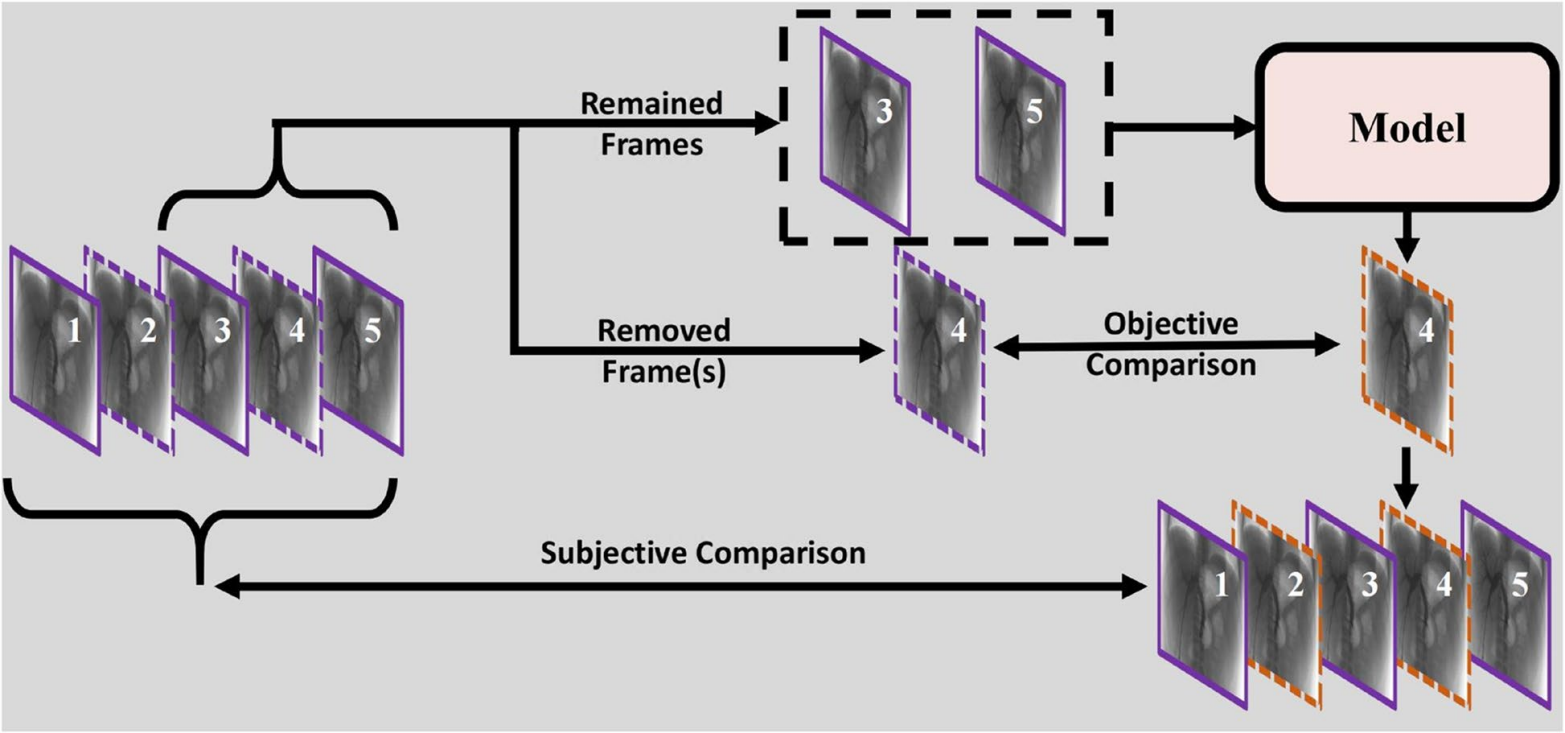
Data flow pipeline for our study. Here, we take a DSA sequence with five frames as an example to illustrate the data processing scheme. For each sequence, we removed every other frame (but always keeping the 1-st frame). The remaining adjacent (e.g., 3rd and 5th) frames are input into a model to predict the missing intermediate frame (i.e., 4th). We use the objective metrics of PSNR, SSIM, and RMSE to quantitatively compare the closeness of the predicted frames to the real (removed) frames. Finally, the remaining (i.e., 1st, 3rd, and 5th) frames and the predicted (i.e., 2nd and 4th) frames are stacked together into a sequence according to priority for the subjective experiments by interventional radiologists

The SepConv +  + network’s performance was assessed on the DSA frame interpolation test set using both objective metrics and visual quality assessment. While SepConv +  + is a standard deep neural network capable of generalizing across diverse patient characteristics (e.g., age, body size, and sex), this study specifically evaluates its impact on IR sequences with varying modalities and frame rates (Fig. 2). We utilized objective metrics including peak-signal-to-noise-ratio (PSNR), Structural Similarity (SSIM) [19], and root mean square error (RMSE) to quantify the disparity between the interpolated frames and the corresponding frames in the original sequences. Typically, higher PSNR and SSIM values (lower RMSE values) indicate better interpolation performance [8, 20, 21]. Additionally, we conducted a visual quality comparison to corroborate the quantitative findings. Furthermore, a two-sample Kolmogorov–Smirnov (K-S) test [22] was carried out to establish the statistical significance of the comparative results. In addition to this evaluation, we conducted a comparison between SepConv +  + and the state-of-the-art (SOTA) video frame interpolation (VFI) method FLAVR [23]. FLAVR utilizes a 3D-UNet to extract multiple frames from the front and rear for synthesizing intermediate frames. It exhibited strong performance in synthesizing common videos.

**Fig. 3:**
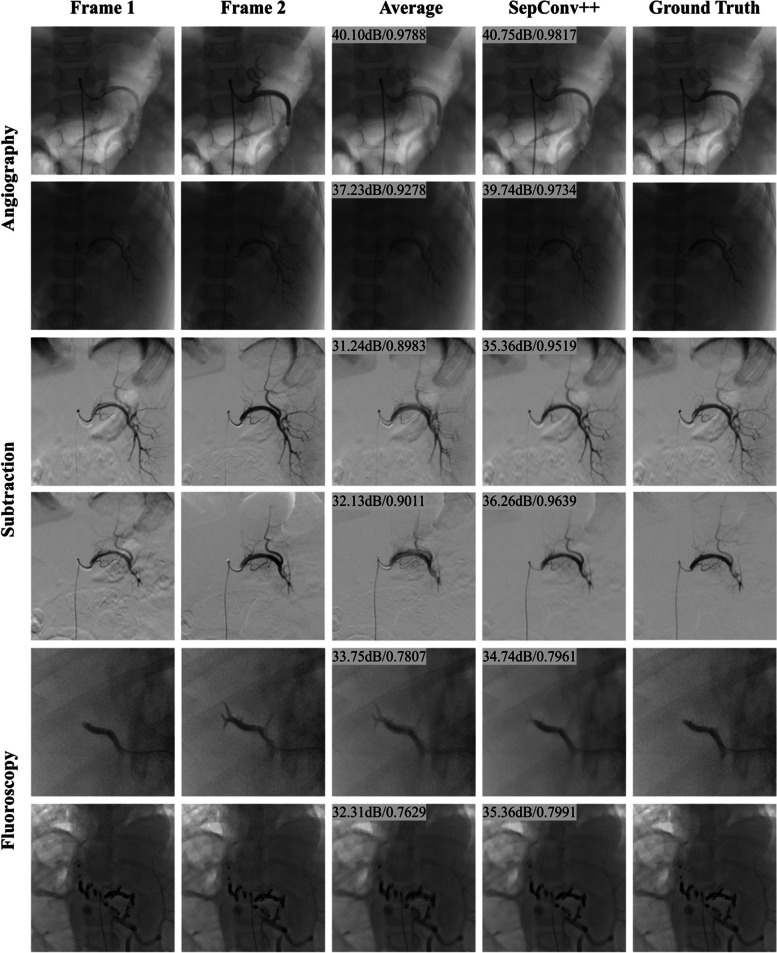
Qualitative and quantitative comparison of the baseline Average and SepConv +  + on three different modalities. The test images are from three modalities, i. e., angiography (1–2 row, redpartial splenic artery embolization image of a patient with megalosplenia), subtraction (3–4 row, partial splenic artery embolization image of another patient with megalosplenia) and fluoroscopy (5–6 row, redhepatic arteriogram image of a patient with liver tumor). The results of PSNR and SSIM by the methods of “Average” (third column) and “SepConv +  + ” (fourth column) are provided for references

**Fig. 4:**
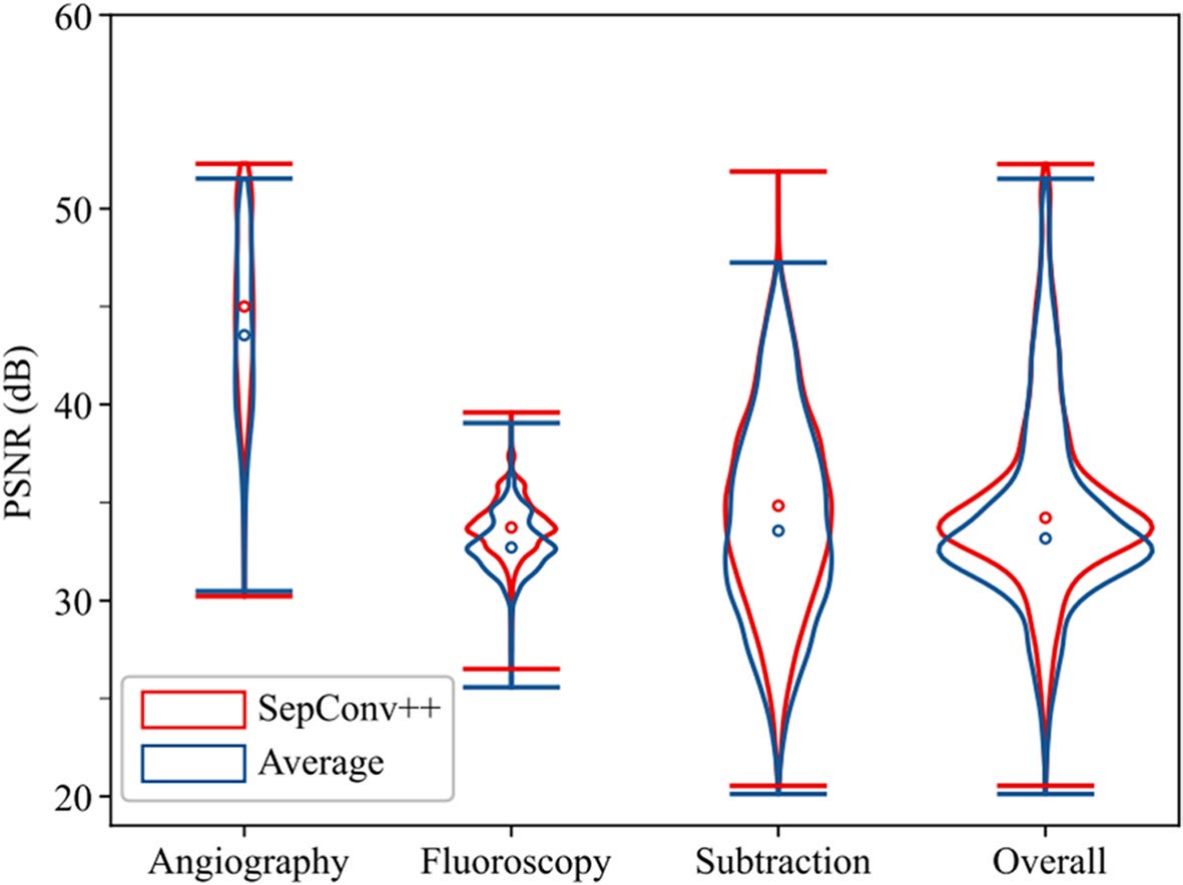
Violin plot results of PSNR distributions in test sets from three modalities and the overall test sets. The width of each violin indicates the density of PSNR results. The total area of each violin represents the number of samples

**Fig. 5:**
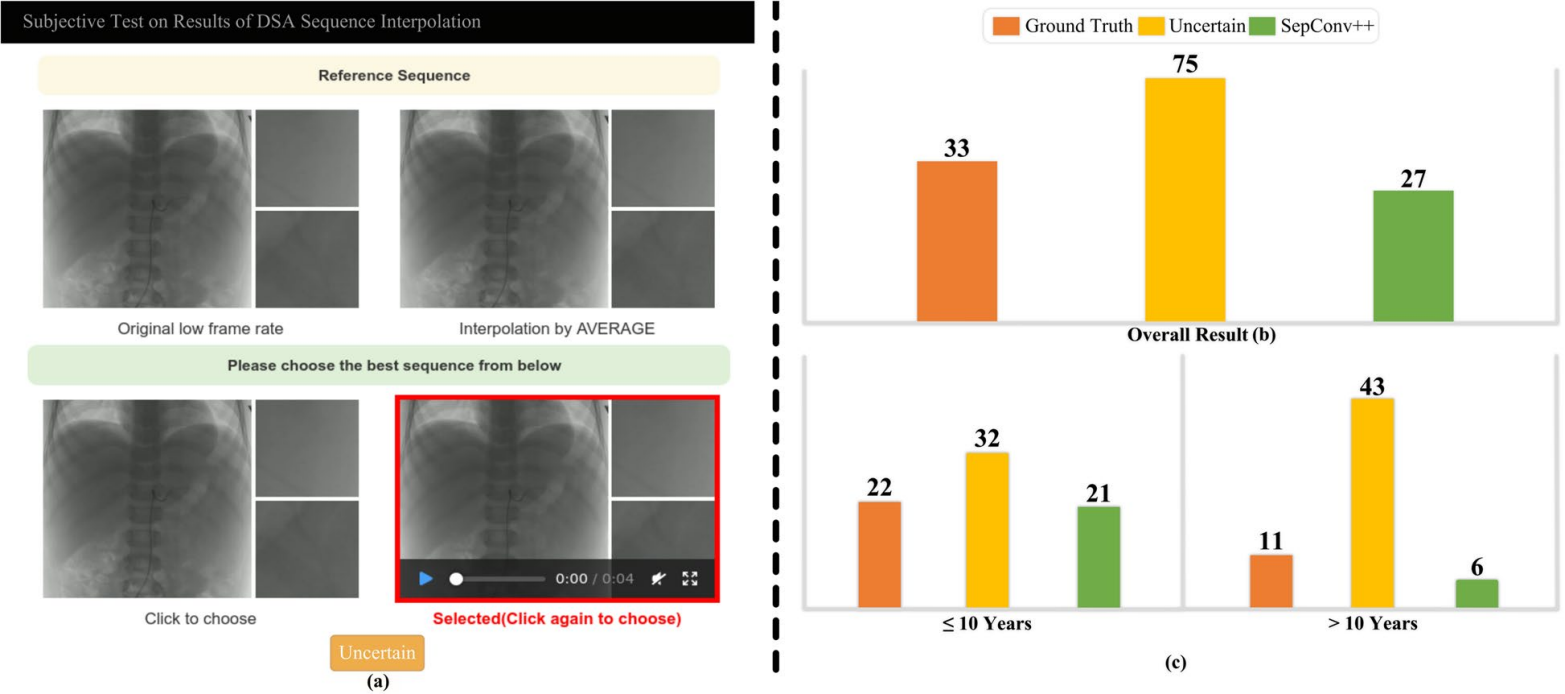
User interface and voting results of subjective experiments on ground truth DSA sequences and the synthetic ones by SepConv +  + . **a** User interface for subjective experiment; **b** the overall voting results; **c** the voting results respect to interventional radiologists’ practice experience in DSA; the red, yellow, and green bars represent votings for “Ground Truth,” “Uncertain,” and the sequences interpolated by “SepConv +  + ,” respectively. The height of each bar represents the corresponding number of votings

To assess the potential clinical application of SepConv +  + , we conducted subjective experiments in a controlled environment involving nine professional interventional radiologists. Initially, we selected 15 representative IR sequences from the test set to serve as the “ground truths.” These sequences comprised 6 angiography sequences, 6 subtraction sequences, and 3 fluoroscopy sequences. Subsequently, for each sequence, we generated low frame-rate sequences for each sequence by removing every other frame. Next, as a baseline method, we synthesized each removed frame by averaging its two adjacent frames. The resulting frames were labeled as “SepConv +  + ” and “Average,” respectively. During the experiments, the interventional radiologists were presented with quadrants displaying “Input,” “Average,” “Ground Truth,” and “SepConv +  + ” for the aforementioned 15 representative sequences, all simultaneously exhibited. The “Input” and “Average” quadrants served as visual references, while “Ground Truth” and “SepConv +  + ” were provided for comparison (refer to Figs. 3 and 4). In Fig. 5a, each interventional radiologist was prompted to choose the superior option between “Ground Truth” and “SepConv +  + ” based on the visual quality. They also had the option to select “Uncertain” if the candidates appeared comparable. Importantly, the radiologists were unaware of the identities of the candidates beforehand. For credibility, the interventional radiologists participating in the subjective experiments were selected from six hospitals and possessed over five years of working experience (e.g., 5, 5, 5, 8, 10, 15, 16, 16, and 34 years, respectively). Their involvement was unrelated to the interests of this study.

## Results

### Overall performance

In our study to evaluate SepConv +  + (also referred as SepConv +  + All) in IR frame interpolation, we compared its performance with the baseline method “Average,” involving averaging of adjacent frames to produce the middle one. This comparative analysis encompassed IR sequences from angiography, subtraction, and fluoroscopy modalities. Table 2 and Fig. 4 illustrate that SepConv +  + outperforms “Average” across all three modalities. The *p*-values derived from the Kolmogorov–Smirnov (K-S) test comparing the PSNR results of SepConv +  + and “Average” are less than 0.05 for each respective modality, indicating significant differences between the two methods. Similar conclusion was also drawn for other metrics like SSIM and RMSE. Furthermore, in our dataset, SepConv +  + also exhibited superior performance compared to the FLAVR method, as shown in Table 2. The intermediate frames generated by SepConv +  + exhibited a remarkably high visual similarity to the corresponding ground-truth frames, as evidenced by the combination of these metrics. Consequently, these frames can be seamlessly integrated into existing frames for clinical diagnosis. Visual comparison between “Average” and SepConv +  + in Fig. 3 revealed that SepConv +  + effectively restored intermediate frames, whereas “Average” often produced blurry edges. These results preferred SepConv +  + in IR sequence interpolation.

**Table 2:** Comparison of PSNR (dB), SSIM [18], and RMSE results by the baseline Average, FLAVR, and four variants of SepConv +  + trained with DSA sequences in different modalities. “SepConv +  + All,” “SepConv +  + A,” “SepConv +  + S,” and “SepConv +  + F” mean that the SepConv +  + is trained with the DSA sequences of all three modalities, angiography modality, subtraction modality, and fluoroscopy modality, respectively.“↑”(or “↓”) means that higher (or lower) is better. The best and second best results are highlighted in bold and *italics*, respectively

Method		Average	SepConv + + All	**Gain**	*p*-value	FLAVR	SepConv + + A	SepConv + + S	SepConv + + F
Angiography	PSNR↑	43.72	**44.94**	+ 1.22	< 0.01	***44.42***	*44.89*	43.62	44.48
	SSIM↑	0.9786	**0.9840**	+ 0.005		***0.9830***	*0.9838*	0.9818	0.9825
	RMSE↓	1.7636	**1.5348**	− 0.228		***1.6299***	*1.5484*	1.7481	1.6130
Subtraction	PSNR↑	33.86	*34.84*	+ 1.01	< 0.01	***34.34***	33.00	**34.86**	33.65
	SSIM↑	0.9028	*0.9194*	+ 0.016		***0.9102***	0.8837	**0.9196**	0.8942
	RMSE↓	4.0441	*3.7446*	− 0.300		***3.8873***	4.2735	**3.7385**	4.1096
Fluoroscopy	PSNR↑	32.81	*33.82*	+ 1.01	< 0.01	***33.72***	33.62	33.16	**33.85**
	SSIM↑	0.7491	*0.7752*	+ 0.026		***0.7736***	0.7691	0.7640	**0.7758**
	RMSE↓	5.3012	*4.8724*	− 0.428		***4.9039***	4.9630	5.1257	**4.8624**

### Performance of SepConv++ on different IR modalities

To evaluate SepConv +  + ’s interpolation performance across different IR modalities, we trained four different variants of SepConv +  + . The initial variant, SepConv +  + All, utilized all IR sequences encompassing the angiography, subtraction, and fluoroscopy modalities. Subsequently, the variants of SepConv +  + A, SepConv +  + S, and SepConv +  + F were exclusively trained on sequences from the angiography, subtraction, and fluoroscopy modalities, respectively. Our examination, using sequences from each individual IR modality, produced insightful results presented in Table 2.

Primarily, SepConv +  + All consistently achieved the highest performance on angiography sequences and obtained the second-best results (close to the best) on the subtraction and fluoroscopy sequences. These results indicate that training SepConv +  + on all IR sequences from the three modalities yielded highly effective frame interpolation with robust generalization capability.

Additionally, training SepConv +  + exclusively with IR sequences from a single modality led to superior performance of the corresponding variant, SepConv +  + X (where “X” represents “A,” “S,” or “S”), on the IR sequences within the respective angiography, subtraction, or fluoroscopy modality. This can primarily be attributed to the presence of domain shift across different modalities, which negatively impacts the generalization ability of SepConv +  + in frame interpolation.

Lastly, the variant SepConv +  + A (or SepConv +  + C) demonstrated superior performance compared to the Average and SepConv +  + S on fluoroscopy (or angiography) sequences. This can be attributed to the closer image style between angiography and fluoroscopy sequences compared to subtraction sequences. Consequently, SepConv +  + S exhibited the worst performance on angiography or fluoroscopy sequences.

### Subjective experiments

The subjective experiments depicted in Fig. 5 demonstrate minimal disparity in voting between the “Ground Truth” (33 votes) and “SepConv++” (27 votes), with a majority of votes categorized as “Uncertain” (75 votes). These results strongly suggest a remarkable similarity between the generated images and real ones, supported by a *p*-value < 0.05. These consistent findings persist across all modalities, frame rates, and among radiologists with varying years of experience (as illustrated in Table 3 and Fig. 5b). Notably, even with the reference of ground truth, physicians were unable to discern any notable differences, suggesting that the interpolated frames possess both high diagnostic and visual qualities. Consequently, SepConv++ holds promising potential for utilization in clinical procedures.

**Table 3:** Interventional radiologists voting on DSA sequences for different modalities, FPS, and work experience

Vote		Ground truth	Uncertain	SepConv + +	*p*-value
Modalities	Angiography	12	32	10	0.7870
	Subtraction	16	27	11	
	Fluoroscopy	5	16	6	
FPS	4 FPS	27	53	19	0.7675
	10 FPS	4	17	6	
	30 FPS	2	5	2	
Experience	< 10 year	41	19	15	0.9614
	> 10 year	34	14	12	

Furthermore, to gain deeper insights, we present the findings in Table 3 and conduct a chi-square test to examine the correlation between voting and the modality, the FPS of IR sequences, as well as working experience of radiologists. In the test of independence between voting and modality, FPS and work experience, respectively, the *p*-value was 0.787, 0.768, and 0.961. It leads to the acceptance of the original hypothesis and indicates the independence of voting results from these factors. In summary, the synthetic IR sequences interpolated by SepConv++ exhibit a striking resemblance to ground truth sequences concerning visual quality and blood flow across various modalities and FPS.

## Discussion

Both interventional and diagnostic imaging procedures involve the use of ionizing radiation, which can induce structural damage at the cellular or molecular level and potentially lead to DNA damage, thereby increasing the risk of cancer [24]. The susceptibility of tissues to radiation-induced damage varies depending on their rate of cell proliferation and the extent of cell differentiation. Consequently, hematopoietic (lymphoproliferative) organs, characterized by rapid cell turnover, exhibit higher sensitivity, whereas neural tissues with minimal or no cell renewal demonstrate lower sensitivity. As a result, children, who constitute a particularly vulnerable group due to their heightened sensitivity to radiation, are at a substantially greater risk of harm from equivalent radiation exposure compared to the adults, and this risk persists for a longer duration.

The utilization of interventional radiology has witnessed a significant increase for both diagnostic examination and treatment, aiming to enhance diagnostic accuracy and reduce the need for invasive procedures [25]. However, despite the extensive discussion surrounding the immediate and long-term benefits of interventional examinations and treatments in children, the associated risks are frequently overlooked. Children exhibit a higher vulnerability to ionizing radiation compared to the adults, with pediatric patients displaying increased radiosensitivity, particularly in tissues such as the thyroid, gonads, and bone marrow. Furthermore, due to their smaller stature, children receive higher radiation doses than adults [26]. Moreover, children have a longer post-radiation exposure survival period and face an extended duration of radiation-induced cancer risk, thereby experiencing a more severe radiation risk than the adults [27].

The likelihood of long-term effects from ionizing radiation is probabilistic and depends on the total radiation dose, yet the severity of these effects cannot be solely attributed to radiation. Other factors, including environmental and genetic factors, also play a significant role. Radiation-induced cancers, such as myeloma, leukemia, lung, thyroid, breast, bone, and skin cancers, may manifest decades after exposure [28]. Therefore, it is crucial to minimize the exposure to ionizing radiation in children undergoing interventional procedures.

Scattered radiation from the patients is the primary source of radiation exposure to the interventionalist during the intervention [29]. Consequently, interventions aimed at reducing radiation exposure to patients can simultaneously minimize radiation exposure to interventionalists. Conventional radiation protection measures primarily emphasize three aspects: exposure time, distance from the X-ray source, and the use of shielding devices [30, 31]. Due to the growing complexity of anatomical and interventional techniques, DSA imaging systems frequently necessitate longer transmission times to obtain high-quality image sequences. Unfortunately, this prolonged exposure unavoidably leads to increased radiation exposure for both patients and interventionalists [4]. Consequently, it becomes imperative to introduce innovative technical solutions aiming at reducing radiation exposure associated with interventional procedures while preserving the quality of IR images.

In recent years, with substantial advancements in artificial intelligence techniques [14, 15, 32], particularly in deep learning-based image processing, these techniques have found widespread applications in various fields such as video frame interpolation, slow-motion generation, graphic animation, and rendering. Furthermore, an increasing number of researchers are opting to integrate these emerging deep learning image-processing techniques into the conventional medical domain. In this study, we propose a fusion of artificial intelligence’s video interpolation technique with interventional radiology images. The objective is to generate radiological and interventional images with reduced frame rates that can subsequently be reconstructed and restored to high quality without compromising the clinical judgment facilitated by deep learning models. Consequently, by decreasing the acquisition frame rate, it is feasible to reduce the duration of radiation exposure, thereby decreasing the radiation dose to both medical practitioners and patients.

Our study utilizes a retrospective approach to simulate high frame-rate IR sequences. This is achieved by artificially extracting frames at a one-frame interval. Subsequently, we employ a robust deep learning model, i.e., SepConv +  + , capable of interpolating frames to reconstruct the original high frame-rate IR sequences. The results are satisfactory, as SepConv +  + effectively restores the original IR images, as indicated by objective evaluation metrics including PSNR, SSIM, and RMSE. In our experimental results, it was challenging for interventionalists at different levels of expertise to distinguish the generated images from the original images. Additionally, the high frame-rate IR images restored by SepConv +  + were deemed clinically irrelevant.

Our study revealed that the preference for interpolated sequences compared favorably to the ground-truth sequences in angiography and fluoroscopy modalities. However, in subtraction sequences, SepConv +  + received fewer selections than the original sequences. This disparity can be attributed to irregular artifacts present in subtraction sequences, disrupting the smooth transition between adjacent frames in synthetic IR sequences.

Our results underscored the robustness of SepConv +  + across different modalities and frame rates. Moreover, we observed a positive correlation between its interpolation performance and the frame rate of IR sequences. The “SepConv +  + All” model, trained using sequences from all three modalities, consistently demonstrated promising performance across IR sequences in the respective modalities. Furthermore, when SepConv +  + is trained on specific IR modalities, it achieves the optimal interpolation results for IR sequences within the corresponding modality. However, in the case of the angiography modality, the variant “SepConv +  + A” (trained with angiography sequences) exhibited slightly inferior performance compared to “SepConv +  + All.” This suggests that data from the other two modalities slightly contribute positively to the training of SepConv +  + in interpolating angiography sequences. “SepConv +  + All” demonstrates excellent performance on IR sequences across various frame rates and achieves superior interpolation results for IR sequences with higher frame rates.

The study presents several limitations that warrant consideration. Firstly, our IR sequences were obtained from pediatric patients undergoing peripheral interventions at a single imaging center. However, it should be noted that sequences obtained from different medical institutions may exhibit variations in image quality due to differences in parameter settings (e.g., voltage and image resolution) of IR imaging devices. The applicability of our deep learning model requires further verification on sequences obtained from diverse imaging devices. Additionally, as different patient populations (adults or pediatrics) exhibit distinct blood flow conditions, the transferability of models trained on pediatric datasets to adults requires investigation and is worth of study. Our ultimate research goal is to develop a model applicable across various modalities, patient demographics, and lesion locations. Secondly, our study did not include IR sequences with significant motion, such as those used in cardiac interventions. While abdominal interventions may involve breathing motion, it is considerably less pronounced compared to the motion caused by heartbeats. Consequently, our model cannot guarantee an equally satisfactory performance for cardiac interventions. Further experiments should be conducted along this direction.

Considering these limitations, the primary challenge in the broader clinical application of our work pertains to the domain gap arising from differences in the training set distribution and real-world scenarios. Variances in imaging settings, patient demographics (e.g., age, gender), and motion levels contribute to this disparity. While our method exhibits high adaptability and generalization in our experiments, we anticipate a slightly diminished performance in real-world scenarios. Nevertheless, we expect acceptable visual quality, leveraging knowledge gained from other modalities.

In conclusion, we have utilized an effective deep learning model for accurate frame interpolation in IR sequences from abdominal interventions. This model holds the potential for reducing radiation doses during IR examinations and treatments for both patients and interventional staff. Our future work will involve evaluating the model’s performance through clinical animal trials to quantify radiation dose reduction, aiming to establish reliability for future interventional radiology procedures.

## Data Availability

The image data used to train and test the artificial intelligence model are not shareable under the current agreement with the data custodian. The derived data, including the model and de-identified human reader classification outputs for the test data (as well as the related data dictionary), will be made available immediately upon publication to anyone who wishes to access the data. Requests for access can be made to the corresponding authors.

## Abbreviations

DSA: Digital subtraction angiography
FPS: Frames per second
IR: Interventional radiology
K-S test: Kolmogorov-Smirnov test
PSNR: Peak signal-to-noise ratio
RMSE: Root mean square error
SOTA: State-of-the-art
SSIM: Structural similarity index
VFI: Video frame interpolation
